# Plasma metabolites associated with biopsychosocial parameters in overweight/obese women with severe knee osteoarthritis

**DOI:** 10.3389/fcell.2024.1454084

**Published:** 2024-09-04

**Authors:** Fabiola Socorro Silva Lisboa, Enzo Martins Benevento, Luisa Oliveira Kaneko, Vanessa Bertolucci, Álex Ap. Rosini Silva, André Cabral Sardim, Valter Ferreira Ruiz, Ivan Gustavo Masseli dos Reis, Andreia M. Porcari, Leonardo Henrique Dalcheco Messias

**Affiliations:** ^1^ Research Group on Technology Applied to Exercise Physiology—GTAFE, Health Sciences Postgraduate Program, São Francisco University, Bragança Paulista, Brazil; ^2^ Research Group on Musculoskeletal Rehabilitation, São Francisco University, Bragança Paulista, Brazil; ^3^ MS4Life Laboratory of Mass Spectrometry, Health Sciences Postgraduate Program, São Francisco University, Bragança Paulista, Brazil

**Keywords:** osteoarthritis, metabolomics, biopsychosocial parameters, knee, woman

## Abstract

**Introduction:**

Obesity aligned with quadriceps muscle weakness contributes to the high incidence of knee osteoarthritis (KOA), which is prevalent in women. Although molecular signatures of KOA have been suggested, the association between biopsychosocial responses and the plasma metabolomic profile in overweight/ obese women with KOA remains in its early stages of investigation. This study aims to associate the plasma metabolome with biopsychosocial parameters of overweight/obese women diagnosed with KOA.

**Methods:**

Twenty-eight overweight/obese women (Control-n = 14; KOA-n = 14) underwent two visits to the laboratory. Functional tests and questionnaires assessing biopsychosocial parameters were administered during the first visit. After 48 h, the participants returned to the laboratory for blood collection. Specific to the KOA condition, the Numerical Pain Rating Scale (NPRS), Tampa Scale for Kinesiophobia (TSK), and Knee injury and Osteoarthritis Outcome Score (KOOS) were applied

**Results:**

Thirteen molecules were different between groups, and four correlated with KOA’s biopsychosocial parameters. DG 22:4-2OH and gamma-Glutamylvaline were inversely associated with KOSS leisure and TSK score, respectively. LysoPE 18:0 and LysoPE 20:5 were positively associated with KOSS symptoms and TSK score, respectively.

**Discussion:**

While the correlations of LysoPE 18:0 and gamma-Glutamylvaline are supported by existing literature, this is not the case for DG 22:4-2OH and LysoPE 20:5. Further studies are recommended to better elucidate these correlations before dismissing their potential involvement in the biopsychosocial factors of the disease.

## Introduction

Osteoarthritis (OA) is a chronic rheumatic disease affecting synovial joints (Jang et al., 2021). OA encompasses pathological changes in the articular cartilage integrity, subchondral bone, joint margins, and overall joint function (Kolasinski et al., 2019). The knee joint is commonly affected by OA, characterized as knee osteoarthritis (KOA) (Jiang et al., 2012; Spinoso et al., 2018). The global prevalence of this disease for adults over 40 years is 19% and the incidence increases with age, while the prevalence in women is 11.9% higher than in men (Cui et al., 2020).

Weakness in the muscles surrounding the knee joint, particularly the quadriceps femoris, directly impairs daily activities by reducing shock absorption during walking and altering gait patterns (Clermont and Barden, 2016; Kierkegaard et al., 2015). External trauma, advanced age, systemic inflammation, and obesity also intensify the KOA symptoms. A meta-analysis suggests a dose-response relationship between obesity and KOA risk; for every 5-unit increase in body mass index (BMI), there is a 35% increased risk (Jiang et al., 2012). Furthermore, physical tests are crucial for clinical decisions regarding KOA. These tests facilitate interventions precisely tailored to the unique needs of each patient. In this sense, one maximum repetition test (1-RM), 30-second sit and stand test (STS), Timed Up and Go test (TUG), and 6-minute walk test (6MWT) are recommended by the Osteoarthritis Research Society International (OARSI) (Bannuru et al., 2019).

Given the complexity and heterogeneity of KOA pathogenesis, molecular signatures emerge to study the pathological condition, drug responses, and early identification of the disease (Zhai et al., 2018). In this context, technological advances like metabolomics have gained considerable space in the scientific community (Bujak et al., 2015; Zhang et al., 2012), allowing the identification of a vast number of metabolites present in organic matrices (e.g., blood, urine, or saliva) (Sakaguchi et al., 2019). A clinical trial found a higher proportion of histidine in KOA patients, suggesting it as a possible biomarker (Zhai et al., 2010). Further evidence suggests energy metabolic profile (glucose metabolism, tricarboxylic acid cycle, and ß-oxidation pathway), lipid metabolism, or amino acid metabolism (Zhai, 2019) modulations in patients with this disease. OA is also related to changes in amino acid metabolism, like arginine and alanine, as well as phospholipids, which have been identified as potential biomarkers to distinguish patients with KOA from healthy patients (Haartmans et al., 2021; Pousinis et al., 2020).

The multifactorial characteristic of KOA along with its higher prevalence in overweight/obese women can increase biopsychosocial symptoms, including chronic pain. Pain is primarily the result of joint dysfunction, progressing to functional incapacity and increasing the level of anxiety, depression, pain catastrophizing, and kinesiophobia (Kopp et al., 2021), making these factors prime targets in the treatment of patients with KOA (Vogel et al., 2022). While molecular signatures were suggested for chronic pain (Russo et al., 2020; Teckchandani et al., 2021), the association of biopsychosocial responses with plasma metabolites of overweight/obese women with KOA was not investigated. This analysis allows for a deeper exploration of the physiological aspects impacted by KOA and the biopsychosocial responses of these patients, opening new avenues for understanding the disease.

This study aims to associate the plasma metabolome of overweight/obese women diagnosed with KOA with biopsychosocial parameters. To this end, the molecular signatures of KOA patients will be compared with a control group, aiming to identify differential molecules. We hypothesize that metabolomics will provide molecular signatures correlated with biopsychosocial parameters of overweight/obese women diagnosed with KOA, such as pain catastrophizing and kinesiophobia.

## Material and methods

### Participants and experimental design

Women seeking treatment for KOA at the São Francisco University were invited to participate in the study. Inclusion criteria for the KOA group consisted of: a) KOA diagnosed by an orthopedist and a rheumatologist, considering the American College of Rheumatology criteria (Hochberg et al., 1995), with radiological confirmation of grade IV according to the Kellgren-Lawrence classification (Kellgren and Lawrence, 1957); (b) minimum of 1 year regarding KOA grade IV diagnosis; (c) age between 55–85 years old; (d) pain reported during activities such as stair climbing, kneeling, sitting and standing up, running, and standing for 2 min; (e) moderate levels of pain according to the Numerical Pain Rating Scale (NPRS) (Chanques et al., 2010; Karaborklu Argut et al., 2021). On the other hand, women were excluded if they presented: (a) uncontrolled diabetes mellitus; (b) untreated systemic arterial hypertension; (c) neurological deficits (motor or sensory); d) a history/diagnosis of cancer; (e) fibromyalgia; (f) prior injection of hyaluronic acid; and (g) any other invasive therapies. Regarding the control group, women aged 55–85 years without KOA were recruited, and the same exclusion criteria for group KOA were considered.

The women underwent two visits to the laboratory. Functional tests and questionnaires were administered during the first visit. After 48 h, the participants returned to the laboratory for blood collection. This study was conducted in accordance of Helsinki criteria and approved by the Ethic Committee of the São Francisco University (63939422.2.0000.5514).

### Functional tests

The functional tests applied are usually administered for the population included in this study (Chanques et al., 2010; Karaborklu Argut et al., 2021). Tests were conducted in the same sequence and time of day. Additionally, all volunteers consumed the same breakfast consisting of a banana, a pack of saltine crackers, and 200 mL of orange juice. This meal provided 342 kcal (4.42 g protein, 4.58 g lipids; 73.84 g carbohydrates). The volunteers wore appropriate clothing for physical exertion and had free access to water consumption during the tests.

The STS assessed lower limb strength and endurance. Beginning from a seated position, the individual repeatedly stood up and sat back down for 30 s, keeping the arms crossed over the chest throughout the test (Boonstra et al., 2008). The TUG evaluated functional mobility, balance, walking capacity, and fall risk. At the researcher’s request, the volunteer was instructed to use their upper limbs to rise from the chair, walk around a cone positioned 3 m away, return to the chair, and sit back down. Human assistance was not permitted at any point during the test. The test was performed twice, and the shortest time obtained was considered (Kraemer et al., 2004).

The 6MWT assessed cardiorespiratory fitness and served as a predictor of aerobic capacity. In a 30-meter corridor, the volunteer walked as quickly as possible for 6 min, with the total distance covered during this time recorded. The 1-RM for knee extensors was conducted using the leg extension machine. The protocol began with a warm-up using minimal load on the machine, followed by 10 repetitions and a 2-minute rest before starting the test. During the test, the individual performed repetitions at half of the machine’s maximum load, continuing until concentric failure (i.e., the inability to complete the movement through the full range of motion). The individual’s 1-RM was determined as previously suggestedBaechle and Earle (Baechle and Earle, 2008).

### Questionnaires for biopsychosocial parameters assessment

The NPRS assessed pain intensity, ranging from 0 to 10, where 0 corresponded to the absence of pain and 10 to the worst imaginable pain (Karaborklu Argut et al., 2021). To evaluate kinesiophobia, the Tampa Scale for Kinesiophobia (TSK) was administered (Siqueira et al., 2007). This questionnaire is self-administered and contains 17 questions that address pain and intensity of symptoms. The score varies from 17 to 68 points, with the higher the score, the greater the degree of kinesiophobia. The Knee injury and Osteoarthritis Outcome Score (KOOS) is a validated self-report questionnaire designed for individuals with KOA. It assesses symptoms, pain, joint stiffness, daily activities, sports and leisure activities, and quality of life (Goncalves et al., 2010). Each subscale is scored separately, with scores ranging from 0 to 100. Higher scores indicate better knee function and lower levels of symptoms or impairment. These questionnaires were applied only to the KOA group.

The World Health Organization Quality of Life-Bref (WHOQOL-Bref) assessed the individual’s quality of life across the physical health, psychological health, social relationships, and environmental domains (Fleck et al., 2000). The instrument consists of 26 items, including two general items about overall quality of life and general health. Each item is rated on a five-point Likert scale, with responses ranging from “very dissatisfied/very poor” to “very satisfied/very good” or “not at all/extremely” to “an extreme amount.” The score for each item within the respective domain is summed and then transformed to a scale of 0–100.

The Epworth Sleepiness Scale (ESS) assessed daytime sleepiness (Bertolazi et al., 2009). The final score is calculated by summing the scores of eight questions, each rated on a scale from 0 to 3. The final score ranges from 0 to 24, with higher scores indicating greater daytime sleepiness. The Pittsburgh Sleep Quality Index (PSQI) is calculated by scoring seven components of sleep quality, including sleep latency, duration, habitual sleep efficiency, disturbances, use of sleeping medication, and daytime dysfunction (Bertolazi et al., 2011). Each component is scored from 0 to 3, and the sum of all component scores yields a total score ranging from 0 to 21, with higher scores indicating poorer sleep quality. Lastly, the Profile of Mood States (POMS) is calculated by assessing mood states (tension, depression, anger, vigor, fatigue, and confusion) through adjective items (Curran et al., 1995). Participants rate how they have felt over the past week on a scale ranging from 0 to 4. The total score is obtained by summing the ratings across multiple mood dimensions. Higher scores in dimensions indicate greater intensity of the mood states.

### Blood sample collection

Participants were instructed by researchers to abstain from alcohol consumption or unusual foods or beverages for 3 days preceding blood collection. A skilled nurse collected 5 mL of venous blood for metabolomic analyses using tubes containing Ethylenediaminetetraacetic acid (EDTA). Following collection, the samples underwent centrifugation at 1.500 rpm for 10 min. Subsequently, plasma was carefully collected by an experienced researcher and stored at −80°C for subsequent metabolomic analysis. Regarding hematological analyses, another 5 mL of venous blood using tubes containing EDTA was collected, and hematological parameters determined by the Pentra 80 (California, United States).

### Metabolites extraction

Before extraction, a 20 μL aliquot from each sample was reserved to create a pooled sample, which served as a quality control (QC). QC samples were interspersed every 10 samples to monitor any deviations in extraction and system stability. Sample extraction and analysis were conducted randomly to minimize instrumental and biological biases. Metabolites from plasma (180 μL) were extracted using cold methanol (380 μL). After vortexing (30 s), the tubes underwent centrifugation (12,000 RPM, 10 min at 4°C). The resulting lower organic layer (420 μL) was collected and dried under N_2_ flow. Dried samples were reconstituted in 150 μL of a solution comprising acetonitrile (ACN): H_2_O (1:1, % v/v).

### Untargeted metabolomics

Untargeted metabolomics analyses were adapted from established methodologies (Silva et al., 2020). An ACQUITY UPLC system coupled with a XEVO-G2XS Quadrupole Time-of-Flight (QToF) mass spectrometer (Waters, Manchester, UK) equipped with an Electrospray Ionization (ESI) source operating in both positive (ESI+) and negative (ESI-) ionization modes was utilized. Chromatographic separation was performed using an ACQUITY UPLC^®^ CSH C18 column (2.1 mm × 100 mm x 1.7 μm, Waters), with mobile phases composed of water with 0.1% formic acid (A) and acetonitrile with 0.1% formic acid (B), flowing at a rate of 0.4 mL min^−1^. The column gradient transitioned from 10% to 40% B over 0.5 min, then to 90% B over 4.5 min, and maintained for 2 min before reverting to 10% B. The system was equilibrated for 2 min, resulting in a total run time of 13 min. Injection volumes were set at 1 μL for positive and negative ion modes.

The mass spectrometer was operated in MS^E^ mode within a m/z range of 50–1,200 Da, with an acquisition time of 0.5 s per scan. MS^E^ analysis utilized a collision energy of 6 V for low energy and a ramp of 20–50 V for high collision energy. Leucine enkephalin was used as the lock mass for mass accuracy, and a 0.5 mM sodium formate solution provided mass calibration. Additional parameters included a source temperature of 135°C, desolvation temperature of 550°C, desolvation gas flow of 900 L h^-1^, capillary voltage of 3.2 kV (ESI+)/2.8 kV (ESI-), and cone voltage of 40 V.

Raw data processing employed Progenesis™ QI v2.4 software (Nonlinear Dynamics, Newcastle, UK) for adduct selection, peak alignment, deconvolution, and compound annotation based on MS^E^ experiments. The software considered adducts such as [M + H]^+^ [M + K]^+^ [M + Na]^+^, and [M + H-H_2_O]^+^ for the positive acquisition mode, and [M-H]^-^ [M + Cl]^-^ [M-H_2_O-H]^-^, and [M + FA-H]^-^ for the negative acquisition mode. Each sample yielded an intensity table of ions, labeled by retention time and nominal masses, known as ‘features’, based on their intensities retrieved from the extracted ion chromatogram areas.

Data processing with Progenesis™ QI v2.4 software involved metabolite identification through fragmentation score, mass accuracy, and isotope similarity, ensuring mass errors ≤5 ppm for the precursor ions and ≤10 ppm for the fragments. External spectra libraries including LipidMaps, Human Metabolome Database, and MoNA - MassBank of North America were consulted. For enhanced fragment matches and compatibility with external libraries, the in-house-developed open-source tool “SDF2PQI” was utilized (Sanches et al., 2023). Finally, the physiological role of the identified molecules was determined using the mentioned libraries and literature papers.

### Statistical analyses

Data is presented as mean and standard deviation (SD). Confidence Intervals (CI) were calculated for SD. The MetaboAnalyst 6.0 environment was adopted for the metabolomics analysis. Molecules were filtered based on technical repeatability QC samples using the Relative Standard Deviation (RSD) greater than 25%. The dataset was median-normalized, log-transformed, and scaled by Pareto. Partial Least Squares Discriminant Analysis (PLS-DA) was applied to classify and discriminate molecules before and after the ILIB. Cross-validation of the model was verified using the Leave-One-Out Cross-Validation method. Variable Importance in Projection (VIP) was assessed considering molecules with a VIP score of ≥1 (Mehmood et al., 2012; Chong and Jun 2005). The comparison between groups of the molecules with VIP score of ≥1 was conducted using an unpaired *t*-test corrected by the False Discovery Rate (FDR) approach. Subsequently, these molecules were subjected to further correlations. Pearson product-moment correlation between the identified molecules and the results from NPRS/TSK/KOOS was performed for the KOA group. In every case, statistical significance was fixed at 5%.

## Results

**FIGURE 1:**
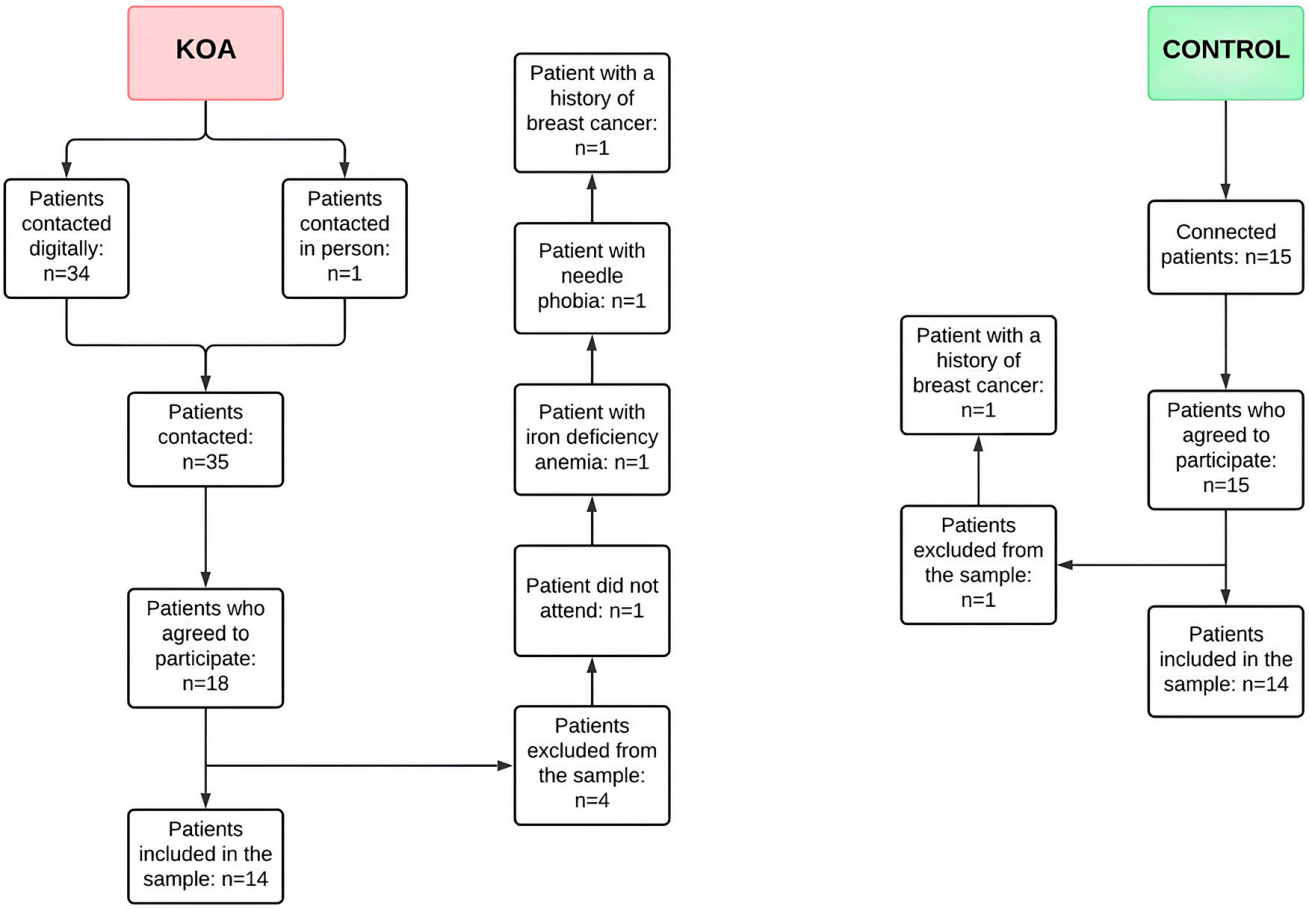
Flowchart containing the recruitment and sample inclusion steps considered in this study. KOA - Knee osteoarthritis.

**FIGURE 2:**
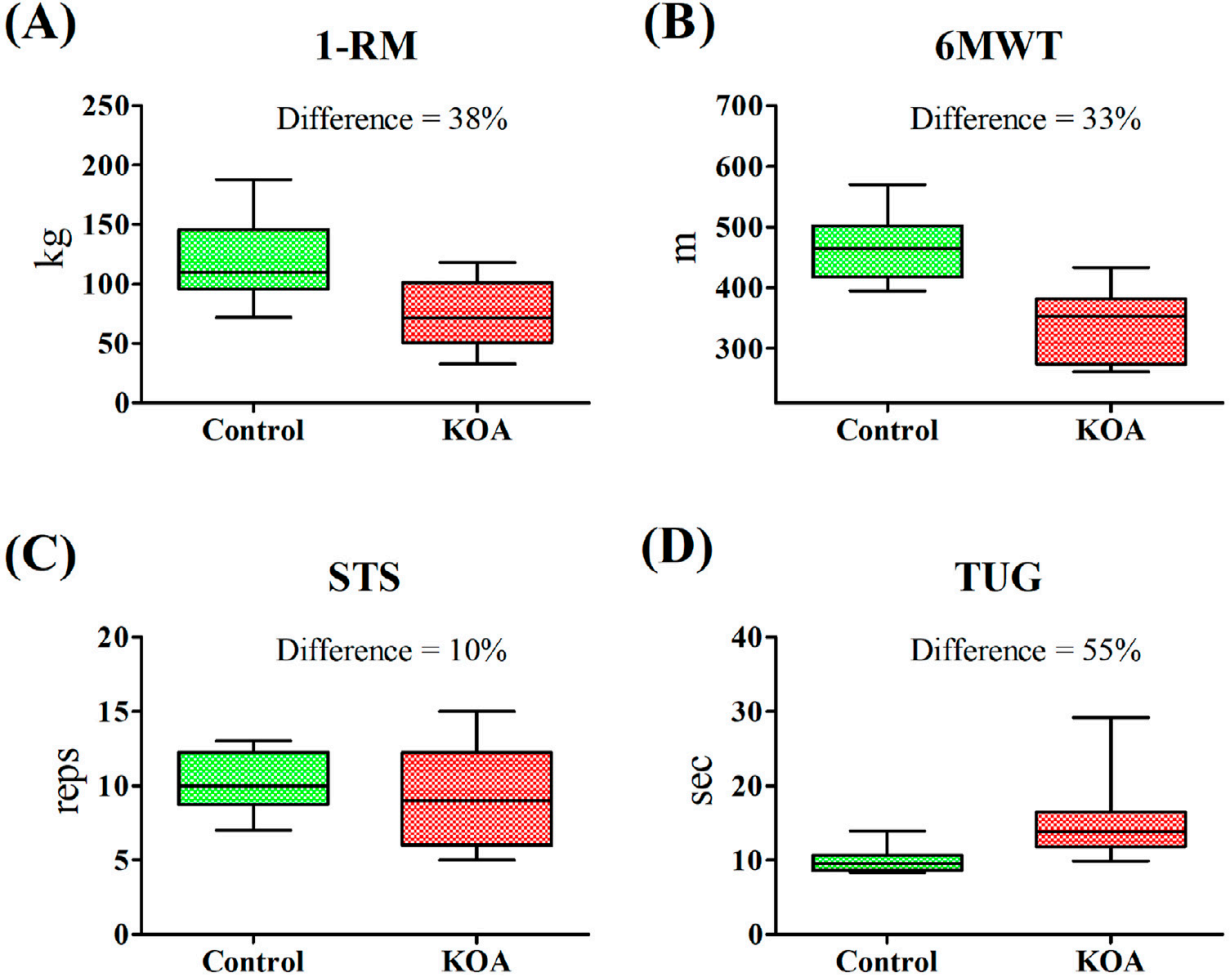
Box and whiskers plot and percentage difference between the control and Knee Osteoarthritis (KOA) groups in functional tests. **(A)** 1-RM—1 maximum repetition test; **(B)** 6MWT—6-minute walk test; **(C)** STS—Sit To Stand test; **(D)** TUG—Time Up and Go.

Twenty-eight women were enrolled in this study, comprising fourteen diagnosed with unilateral or bilateral KOA and fourteen controls (Figure 1). Comparison between the experimental groups regarding age, height, body mass, functional, sleep, psychological, and hematological parameters are shown in Table 1. Controls presented higher performance on 1-RM, 6MWT, and TUG compared to the KOA group, while higher BMI was obtained for the latter group. Figure 2 presents the maximum and minimum values and the percentage difference in the results of the functional tests. Consistent with the statistical difference shown in Table 1, patients diagnosed with KOA exhibited 38% lower strength compared to the controls. Regarding cardiorespiratory fitness, a 38% lower performance in the 6MWT was also observed for KOA patients. In the TUG test, a 55% worse performance was obtained for KOA patients compared to the controls.

**TABLE 1:** Comparison between the experimental groups regarding age, height, body mass, functional, sleep, psychological, and hematological parameters.

	Control	KOA	*p*	ES
Age (years)	60 ± 5	62 ± 8	0.373	0.35
Heigth (cm)	1.62 ± 0.06	1.58 ± 0.07	0.071	0.71
Body Mass (kg)	77.27 ± 14.04	85.89 ± 13.55	0.120	0.61
BMI (kg m^-2^)	29.4 ± 3.8	34.6 ± 5.4	0.007*	1.11
1-RM (kg)	121 ± 43	75.83 ± 26.99	0.000*	1.51
6MWT (m)	467.92 ± 56.53	315.83 ± 102.22	0.006*	1.92
STS (reps)	10.29 ± 2.05	9.29 ± 3.29	0.343	0.37
TUG (sec)	9.82 ± 0.75	15.23 ± 4.71	0.002*	1.98
WHOQOL-Bref (score)	9.87 ± 1.09	10.58 ± 2.25	0.297	0.42
Sleep Quality (score)	7.54 ± 3.28	9.38 ± 3.15	0.156	0.57
ESS (score)	7.18 ± 5.90	6.00 ± 4.77	0.611	0.22
POMS (score)	118.14 ± 25.43	129.71 ± 25.54	0.240	0.45
RBC (10^6^/ul)	4.4 ± 0.25	4.6 ± 0.50	0.390	0.39
Hemoglobin (g/dL)	13.7 ± 0.6	14.0 ± 1.0	0.322	0.43
Hematocrit (%)	40.6 ± 2.0	41.4 ± 2.9	0.479	0.31
MCV (fL)	91.5 ± 2.2	91.2 ± 7.4	0.871	0.08
MCH (pg)	30.8 ± 0.8	30.8 ± 2.5	0.990	0.01
MCHC (10^6^/ul)	33.6 ± 0.28	33.9 ± 0.50	0.176	0.62
RDW (%)	14.4 ± 0.77	14.9 ± 1.5	0.288	0.49
Platelet (10^9^/L)	264.64 ± 76.42	256.54 ± 102.89	0.793	0.11
WBC (10^9^/ul)	7.145 ± 1.819	7.183 ± 1.762	0.960	0.02
Segmented (10^9^/L)	4.383 ± 1.188	4.445 ± 1.269	0.905	0.05
Eosinophils (10^9^/L)	2.052 ± 1.237	1.716 ± 477	0.392	0.39
Lymphocytes (10^9^/L)	2.059 ± 618	2.122 ± 477	0.785	0.12
Monocytes (10^9^/L)	442 ± 206	444 ± 175	0.980	0.01

KOA, Knee osteoarthritis; ES, Effect size; BMI, Body mass index; 1-RM, 1 maximum repetition test; 6MWT, 6-minute walk test; STS, Sit To Stand test; TUG, Time Up and Go; WHOQOL-Bref - World Health Organization Quality of Life-Bref; POMS, Profile of Mood States; ESS, Epworth Sleepiness Scale; RBC, Red Blood Cells; MCV, Mean corpuscular volume; MCH, Mean corpuscular; MCHC, Mean corpuscular hemoglobin concentration; RDW, Red cell distribution width; **WBC**, White Blood Cells; Segmented - segmented neutrophils; *p < 0.05.

**FIGURE 3:**
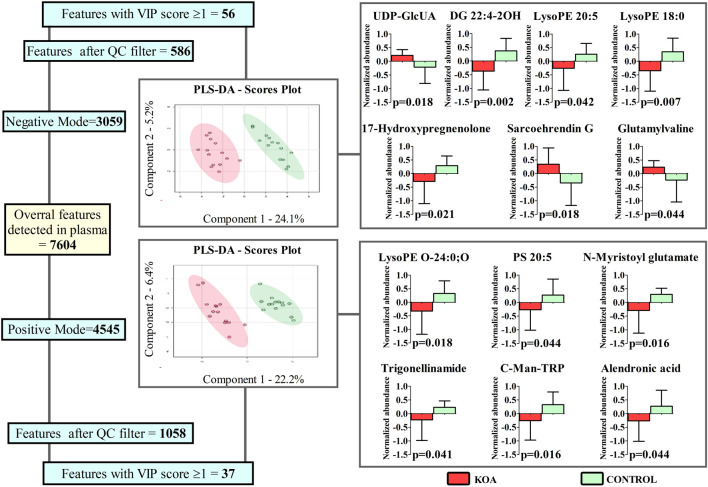
Features detected, filtered by the Quality Control (QC) samples, identified, and compared between women with (KOA) and without (Control) Knee Osteoarthritis via Partial Least Squares Discriminant Analysis (PLS-DA) and unpaired t-test (graphics in the right); *p < 0.05.

All steps and corresponding results of metabolomics are presented in Figure 3. In total, 93 molecules presented VIP score ≥1, being 56 found in negative ionization mode and 37 in positive ionization mode. In both cases, PLS-DA exhibited high validation indices (Negative - Accuracy = 1.000; R2 = 0.874; Q2 = 0.822; Positive - Accuracy = 0.964; R2 = 0.872; Q2 = 0.808). Out of the 93 molecules, 13 were identified with low mass error (range = −3.49 - 4.50). Details including adducts, formula, mass error, *m/z*, and retention time are presented in Supplementary Table S1. In this file is also presented the consistency of LC-MS analysis, where the PCA analysis reveals distinct groupings of the QC samples (Supplementary Figure S1), showcasing the method’s reliability and reproducibility.

Regardless of the ionization mode, the identified molecules belong to distinct classes. Six are lipid and lipid-like molecules (DG 22:4-2OH, Sarcoehrendin G, LysoPE O-24:0; O, LysoPE 20:5, LysoPE 18:0 and PS 20:5), two Amino acids, peptides, and analogs (N-Myristoyl glutamate and gamma-Glutamylvaline), and the remaining are indolyl carboxylic acids and derivatives (C-Man-TRP), pyridinecarboxylic acids and derivatives (Trigonellinamide), bisphosphonates (Alendronic acid), gamma butyrolactones (UDP-GlcUA), or Sulfated steroids (17-Hydroxypregnenolone sulfate). Excepting from UDP-GlcUA, Sarcoehrendin G, and gamma-Glutamylvaline, reduced abundance was observed for the molecules in the plasma of women with KOA.

**FIGURE 4:**
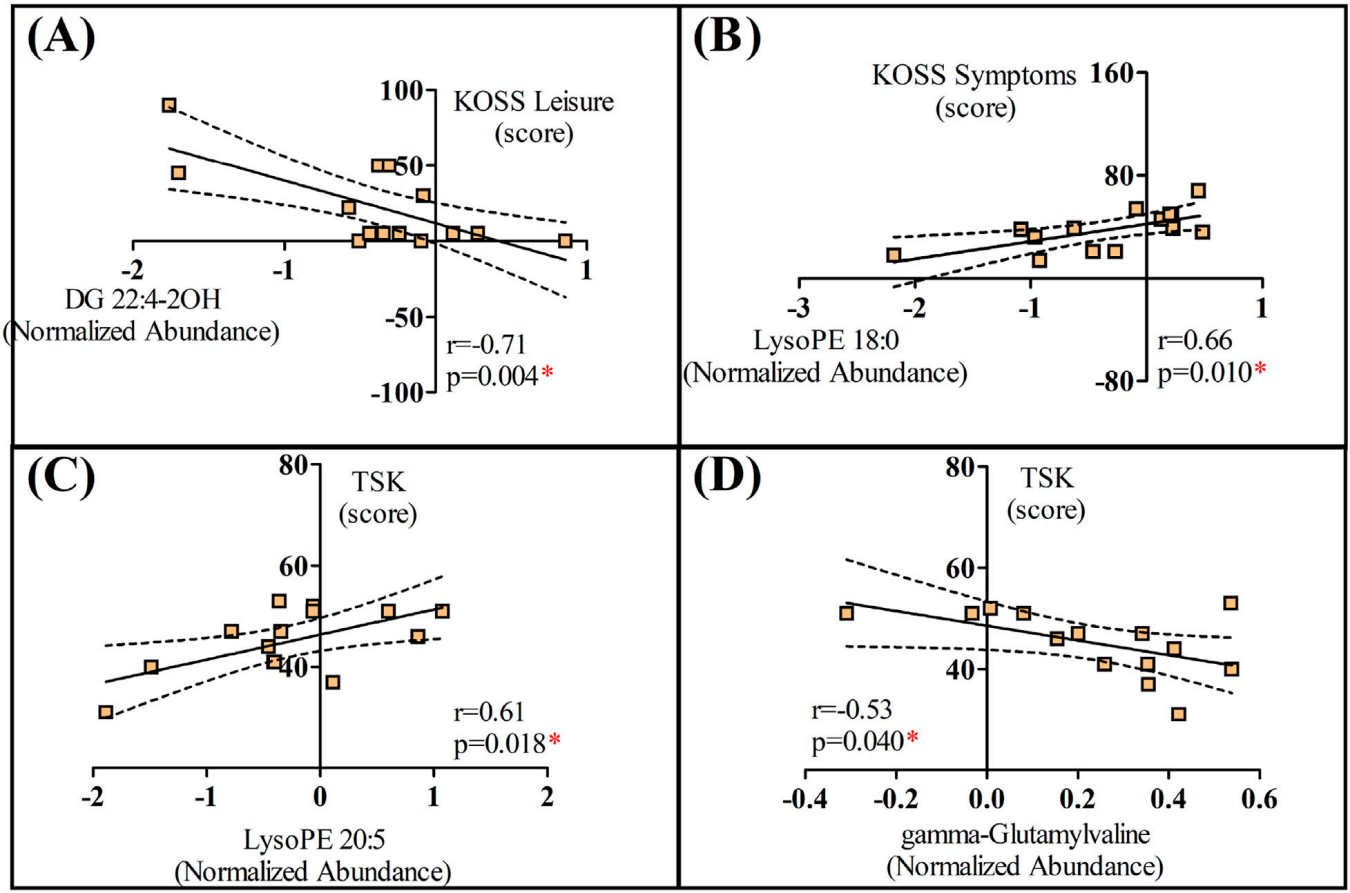
Significant correlations between biopsychosocial parameters and molecules identified in women with knee osteoarthritis (KOA); **(A)** Correlation between the “Leisure” dimension of the Knee injury and Osteoarthritis Outcome Score (KOSS) and DG 22:4-2OH; **(B)** Correlation between the “Symptoms” dimension of the Knee injury and Osteoarthritis Outcome Score (KOSS) and LysoPE 18:0; **(C)** Correlation between the Tampa Scale for Kinesiophobia (TSK) score and LysoPE 20:5; **(D)** Correlation between the Tampa Scale for Kinesiophobia (TSK) score and gamma-Glutamyvaline. *p < 0.05.

NPRS, TSK, and KOOS results from the KOA group are presented in Table 2. Significant correlations were observed for two dimensions of KOA (leisure and symptoms) and the TSK score (Figure 4). While DG 22:4-2OH (Figure 4A) and gamma-Glutamylvaline (Figure 4D) were inversely associated with KOSS leisure and TSK score, respectively, LysoPE 18:0 (Figure 4B) and LysoPE 20:5 (Figure 4C) were positively associated with KOSS symptoms and TSK score, respectively. Given that BMI was higher in the control group, correlations were conducted between this parameter and the molecules that significantly differed between the groups. While Trigonellinamide was correlated with BMI in the control group (r = −0.54; *p* = 0.044), significant correlation between the molecules associated with biopsychosocial parameters regarding KOA group were not observed for controls (BMI and DG 22:4-2OH–r = 0.30; *p* = 0.298; BMI and LysoPE 18:0 – r = 0.09; *p* = 0.736; BMI and LysoPE 20:5 – r = −0.01; *p* = 0.972; BMI and gamma-Glutamylvaline–r = 0.11; *p* = 0.737) (Supplementary Table S2). These results exclude the influence of this parameter on the correlations presented in Figure 4. Likewise, considering that the control group exhibited better performance in most functional tests, the differential molecules were also correlated with these outcomes. However, no significant correlations were observed (Supplementary Table S3).

**TABLE 2:** KOSS, TSK, and NPRS results from the KOA group.

	KOA	Range	CI
KOSS Symptoms (score)	39.80 ± 15.11	14–68	10.95–24.34
KOSS Pain (score)	46.60 ± 6.19	14–64	4.49–9.97
KOSS ADL (score)	43.80 ± 5.54	6–65	4.02–8.93
KOSS Leisure (score)	28.00 ± 22.53	0–90	16.33–36.30
KOSS Quality of Life (score)	21.40 ± 19.42	0.56	14.08–31.29
TSK (score)	43.83 ± 5.31	31–53	3.85–8.55
NPRS (score)	7.82 ± 1.72	3.0–9.8	1.25–2.77

KOA, Knee osteoarthritis; CI, Confidence interval; KOOS, Knee injury and Osteoarthritis Outcome Score; ADL, Activities of Daily Living; TSK, Tampa Scale for Kinesiophobia; NPRS, Numerical Pain Rating Scale.

## Discussion

Our initial hypothesis was that metabolomics would provide molecular signatures correlated with biopsychosocial parameters of overweight/obese women diagnosed with KOA, such as pain catastrophizing and kinesiophobia. The correlations between LysoPE 18:0 and KOSS symptoms as well as gamma-Glutamylvaline and TSK contribute to the existing literature on lipids and amino acids involvement in OA. However, the correlations between DG 22:4-2OH and KOSS leisure or LysoPE 20:5 and TSK suggest these molecules may not be strong markers linking KOA biopsychosocial factors.

The assessment of functional performance in individuals with varying degrees of KOA demonstrates significant biomechanical differences that intensify with disease severity, including gait mechanics (Pollet and Buraschi, 2021). A key distinction is the increased lateral trunk lean during walking observed in individuals with severe KOA. This compensatory mechanism, as emphasized by Hunt et al. (Hunt et al., 2010), likely functions to decrease the load on the affected knee joint, thereby reducing pain, albeit at the expense of imposing additional strain on other parts of the musculoskeletal system. In contrast, this adaptation is less evident in individuals with mild KOA, who tend to maintain a more neutral trunk posture, resulting in more stable and efficient gait mechanics.

The results of this study are consistent with the existing literature. With the exception of the STS test, patients with severe KOA demonstrated poorer performance on functional tests. Further exacerbating the functional impairments in severe KOA, (Sagawa et al., 2017), reported that individuals with advanced KOA exhibit significantly slower walking speeds and greater difficulties with functional tasks. These functional deficits are closely associated with the reduced muscle strength and joint instability characteristic of advanced KOA stages. In contrast, individuals with mild KOA retain better muscle function and joint stability, enabling more effective performance in daily activities. Furthermore, as KOA severity worsens, a progressive decline in muscle strength, particularly in the quadriceps, is also reached. Individuals with this condition experience significant quadriceps weakness, which not only undermines knee joint stability but also intensifies the challenges associated with weight-bearing tasks (Bily et al., 2019).

Although differences in functional tests between patients with severe KOA and individuals without the disease are well established, the association of KOA with biopsychosocial parameters is an emerging area of research. In this context, reinforcing the use of subjective scales to better understand the impact of the disease on these parameters is essential. The KOOS questionnaire evaluates physical function in knee injury patients, assessing mobility, edema, and clicking in the symptom subsection, and activities like running, squatting, and jumping in the leisure assessment subsection. Thus, KOSS symptoms variable is also related to the physical state of the affected limb (Goncalves et al., 2010). Furthermore, the correlation between this parameter and LysoPE 18:0 aligns with the literature on previous studies involving OA, including KOA. Zhang et al., (2016) suggested that the ratio of lysophosphatidylcholines to phosphatidylcholines could serve as a novel metabolic marker for predicting advanced KOA. Indeed, a study on synovial fluid metabolomics has shown that individuals with KOA exhibit higher levels of phospholipids (Kosinska et al., 2013).

LysoPE is classified as a neuronutrient activator through the mitogen-activated protein kinase signaling pathway in pheochromocytoma cells (Nishina et al., 2006; Yamamoto et al., 2022). It has been reported that this lipid stimulates neurite outgrowth and protects against glutamate toxicity in cultured cortical neurons (Hisano et al., 2021a; Hisano et al., 2021b). A previous study showed that LysoPE inhibits lipopolysaccharide-induced M1 macrophage polarization in mouse peritoneal macrophages (Park and Im, 2021). Furthermore, lysophospholipids have been implicated in inflammation and cartilage degradation, which are relevant to KOA. Increased abundances of lysophospholipids may suggest an imbalance in the articular system, contributing to degradation of articular cartilage and leading to KOA progression (Pousinis et al., 2020; Kosinska et al., 2013; Murakami et al., 2017). Researchers also identified that lysophosphatidylcholine and LysoPE may play a role in the KOA pathogenesis, mainly associated with the inflammatory profile (Zhai et al., 2019a; Zhai et al., 2019b). In addition, serum metabolomic analyses in animal models have also identified changes in lysophospholipids metabolism due to KOA (Geraldo et al., 2021; Rocha et al., 2021).

Apart from the fact that these lipids are identified as biomarkers for the disease progression, our results provide new information by demonstrating that LysoPE 18:0 is also associated with biopsychosocial parameters of KOA. The same, however, cannot be transposed for DG 22:4-2OH or LysoPE 20:5. Although both lipids are also involved in phospholipid metabolism, the correlations found with the biopsychosocial parameters of KOA are in opposite directions to the theoretical framework provided so far. These results suggest that not all lipids can be grouped when associated with OA, especially when overweight/obese women. Taking into account the modulation of several lipids’ classes by physiological and pathological stimuli (e.g., aging, sex, exercise, cold exposure, and obesity) on the adipose tissue lipidome (Cho et al., 2023), future studies are recommended before dismissing these molecules concerning their association with KOA biopsychosocial factors.

Kinesiophobia refers to the irrational and excessive fear of movement or physical activity, which can have significant implications in KOA. Mehta et al. (2023) identified amino acids associated with fear of movement in people with symptomatic KOA, which corroborates the observed correlation between gamma-Glutamylvaline and TSK. Such relationships are supported by the amino acid’s roles in health and disease. Amino acids are predictors of general tissue homeostasis (Wu, 2010), besides being irreplaceable as building blocks of proteins and cellular metabolism (Wu, 2009). Additionally, the elevated levels of essential amino acids, such as leucine, are associated with an increased rate of collagen degradation (Zhai, 2019). Further, amino acids regulate metabolic pathways associated with health, survival, growth, development, and reproduction, with some related to chronic pain (Wu, 2013).

This study is not without limitations. Due to the cross-sectional and exploratory design, further inferences regarding the physiological mechanisms underlying the correlations presented in Figure 4 cannot be made at this time. However, the associations found do strengthen the use of the questionnaires employed for understanding the biopsychosocial changes associated with KOA. In this regard, some points warrant emphasis. First, severe KOA indeed induces systemic changes that can be detected using metabolomic techniques. Although mass spectrometry may not be a feasible option in the daily practice of professionals managing KOA, understanding the biopsychosocial impacts of the disease can assist in treatment planning and monitoring, which holds significant clinical value. Thus, our findings contribute to this understanding by demonstrating that commonly used clinical scales are associated with metabolic responses, indicating that these instruments reflect not only the subjective state of the patient, but also linked to the systemic changes induced by severe KOA.

## Conclusion

This study provides new evidence regarding the relationship between metabolomics and biopsychosocial factors of KOA. LysoPE 18:0 and gamma-Glutamylvaline were significantly associated with the biopsychosocial factors of this disease. While the literature supports the importance of these associations, the same cannot be extrapolated to the correlations found for lipids DG 22:4-2OH or LysoPE 20:5 concerning these factors. Future studies are recommended to elucidate these correlations before dismissing their involvement with the biopsychosocial factors of the disease.

## Data Availability

The original contributions presented in the study are included in the article/Supplementary Material, further inquiries can be directed to the corresponding author.
