# Supplementary material for: Plasma metabolites associated with biopsychosocial parameters in overweight/obese women with severe knee osteoarthritis
Source: Front Cell Dev Biol. 2024 Sep 4;12:1454084. doi: 10.3389/fcell.2024.1454084 (PMC11408288; doi:10.3389/fcell.2024.1454084)
Supplement: Supplementary file 1 [file DataSheet1.pdf]

SUPPLEMENTARY FILES

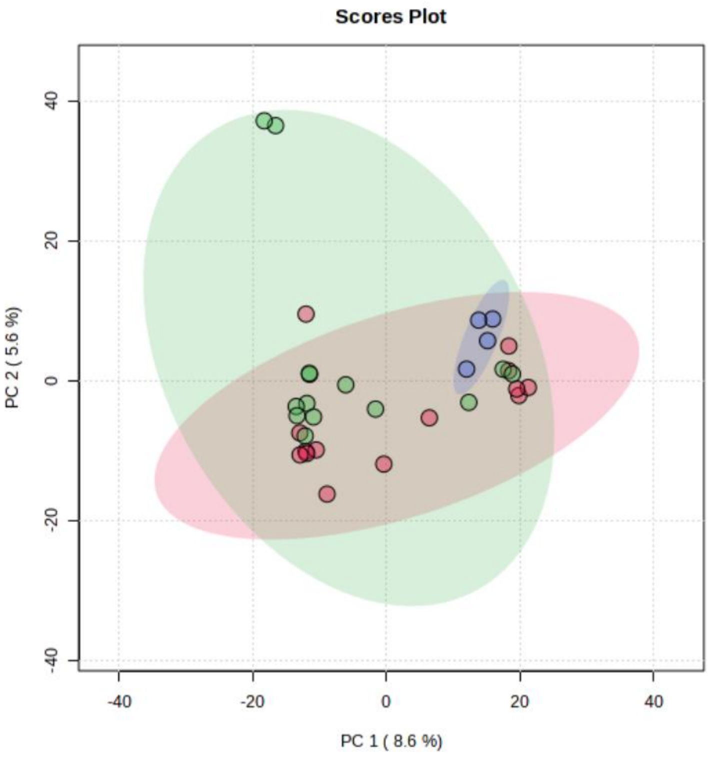

Supplementary Figure 1. Principal Component Analysis (PCA) results. PCA using all features detected. The blue points represent the QC Samples.

Supplementary Table 1. Compounds identified.

m/z measured	Retention Time (min)	Molecular Formula	Adduct	Compound Name	Mass Error (*ppm*)
567,4250	6,55	$C_{29}H_{62}NO_7P$	M+H, M+Na	LysoPE O-24:0;O	-2,46
558,2455	7,54	$C_{26}H_{40}NO_{10}P$	M+H	PS 20:5	-1,42
367,1492	0,79	$C_{17}H_{22}N_2O_7$	M+H	Tryptophan 2-C-mannoside	-2,23
358,2576	4,13	$C_{19}H_{35}NO_5$	M+H	N-Myristoyl Glutamic acid	-3,49
160,0610	1,00	$C_7H_9N_2O$	M+Na	Trigonellinamide	2,07
136,5072	0,86	$C_4H_{13}NO_7P_2$	M+H+Na	Alendronic acid	4,50
580,0352	0,70	$C_{15}H_{22}N_2O_{18}P_2$	M-H, M+Cl	Uridine diphosphate glucuronic acid	1,57
497,2767	4,94	$C_{25}H_{40}O_7$	M+FA-H	DG 22:4-2OH	2,52
411,1844	5,74	$C_{21}H_{32}O_6S$	M-H	17-Hydroxypregnenolone sulfate	-0,60
455,2274	4,97	$C_{22}H_{34}O_7$	M+FA-H	Sarcoehrendin G	-3,03
498,2617	6,16	$C_{25}H_{42}NO_7P$	M-H	LysoPE 20:5	-1,79
291,1191	5,84	$C_{10}H_{18}N_2O_5$	M+FA-H	gamma-Glutamylvaline	-2,74
462,2976	7,39	$C_{23}H_{48}NO_7P$	M-H2O-H	LysoPE 18:0	-2,93

Supplementary Table 2. Correlations of the control group between Body Mass Index and molecules significantly different between the experimental groups.

	r	P
LysoPE O-24:0;O	-0.20	0.496
PS 20:5	0.01	0.974
Tryptophan 2-C-mannoside	0.19	0.496
N-Myristoyl glutamate	-0.05	0.841
Trigonellinamide	-0.54	0.044*
Alendronic acid	0.01	0.974
Uridine diphosphate glucuronic acid	-0.15	0.597
DG 22:4-2OH	0.29	0.298
17-Hydroxypregnenolone sulfate	0.26	0.357
Sarcoehrendin G	0.32	0.258
LysoPE 20:5	-0.01	0.972
gamma-Glutamylvaline	-0.11	0.702
LysoPE 18:0	0.09	0.736

*indicates significant correlation ($p \leq 0.05$).

Supplementary Table 3. Correlations of the control group between functional tests results and molecules significantly different between the experimental groups.

	**1-RM**	**6MWT**	**STS**	**TUG**
LysoPE O-24:0;O	r=-0.52 (p=0.06)	r=0.15 (p=0.604)	r=0.12 (p=0.664)	r=0.12 (p=0.676)
PS 20:5	r=0.34 (p=0.229)	r=-0.26 (p=0.354)	r=0.10 (p=0.714)	r=-0.24 (p=0.398)
Tryptophan 2-C-mannoside	r=0.16 (p=0.577)	r=-0.48 (p=0.080)	r=-0.08 (p=0.777)	r-0.10 (p=0.709)
N-Myristoyl glutamate	r=0.21 (p=0.467)	r=-0.03 (p=0.909)	r=0.06 (p=0.823)	r=-0.29 (p=0.307)
Trigonellinamide	r=-0.52 (p=0.06)	r=0.15 (p=0.604)	r=0.12 (p=0.665)	r=0.12 (p=0.676)
Alendronic acid	r=0.34 (p=0.229)	r=-0.26 (p=0.354)	r=0.10 (p=0.713)	r=-0.24 (p=0.398)
Uridine diphosphate glucuronic acid	r=0.38 (p=0.178)	r=0.10 (p=0.719)	r=0.11 (p=0.694)	r=0.10 (p=0.726)
DG 22:4-2OH	r=-0.01 (p=0.961)	r=-0.17 (p=0.539)	r=0.23 (p=0.426)	r=0.05 (p=0.841)
17-Hydroxypregnenolone sulfate	r=0.41 (p=0.148)	r=-0.03 (p=0.910)	r=-0.49 (p=0.081)	r=0.34 (p=0.227)
Sarcoehrendin G	r=0.31 (p=0.285)	r=0.43 (p=0.116)	r=-0.38 (p=0.180)	r=0.06 (p=0.830)
LysoPE 20:5	r=0.31 (p=0.275)	r=-0.30 (p=0.291)	r=-0.34 (p=0.223)	r=0.21 (p=0.461)
gamma-Glutamylvaline	r=-0.10 (p=0.726)	r=0.41 (p=0.137)	r=-0.22 (p=0.432)	r=0.01 (p=0.996)
LysoPE 18:0	r=0.19 (p=0.517)	r=-0.21 (p=0.457)	r=0.02 (p=0.921)	r=-0.31 (p=0.266)

1-RM - 1 maximum repetition test; **6MWT** - 6-minute walk test; **STS** - Sit To Stand test; **TUG** - Time Up and Go.
